# Dietary, lifestyle and clinicopathological factors associated with *BRAF *and *K-ras *mutations arising in distinct subsets of colorectal cancers in the EPIC Norfolk study

**DOI:** 10.1186/1471-2407-10-99

**Published:** 2010-03-16

**Authors:** Adam Naguib, Panagiota N Mitrou, Laura J Gay, James C Cooke, Robert N Luben, Richard Y Ball, Alison McTaggart, Mark J Arends, Sheila A Rodwell

**Affiliations:** 1 Medical Research Council Dunn Human Nutrition Unit, Wellcome Trust/MRC Building, Cambridge, CB2 0XY, UK; 2 Medical Research Council Centre for Nutritional Epidemiology in Cancer Prevention and Survival, Department of Public Health and Primary Care, University of Cambridge, Cambridge CB1 8RN, UK; 3 Norfolk and Waveney Cellular Pathology Network, Norfolk and Norwich University Hospital NHS Foundation Trust, Colney Lane, Norwich, NR4 7UY, UK; 4 Department of Pathology, University of Cambridge, Addenbrooke's Hospital, Cambridge CB2 2QQ, UK

## Abstract

**Background:**

*BRAF *and *K-ras *proto-oncogenes encode components of the ERK signalling pathway and are frequently mutated in colorectal cancer. This study investigates the associations between *BRAF *and *K-ras *mutations and clinicopathological, lifestyle and dietary factors in colorectal cancers.

**Methods:**

186 adenocarcinomas and 16 adenomas from the EPIC Norfolk study were tested for *BRAF *and *K-ras *mutations. Diet and lifestyle data were collected prospectively using seven day food diaries.

**Results:**

*BRAF *V600E mutation was found in 15.6% of colorectal cancers but at higher frequencies in cancers with proximal location, poor differentiation and microsatellite instability (MSI) (all p < 0.001). *K-ras *mutation (mostly in codons 12 and 13) was found in 22.0% of colorectal cancers but at higher frequencies in cancers of more advanced Dukes' stage (p = 0.001), microsatellite stable (MSS) status (p = 0.002) and in individuals with lower blood high-density lipoprotein concentrations (p = 0.04). Analysis of dietary factors demonstrated no link between *BRAF *mutation and any specific dietary constituent, however, *K-ras *mutation was found at higher frequencies in individuals with higher white meat consumption (p < 0.001). Further analysis of specific mutation type demonstrated that G to A transitions in *K-ras *were observed at higher frequencies in individuals consuming lower amounts of fruit (p = 0.02).

**Conclusion:**

These data support the model of *BRAF *and *K-ras *mutations arising in distinct colorectal cancer subsets associated with different clinicopathological and dietary factors, acting as mutually exclusive mechanisms of activation of the same signalling pathway.

## Background

*BRAF *and *K-ras *genes both encode proteins that act in the ERK signalling pathway, which mediates cellular responses to growth factors and regulates elements of the cell cycle, apoptosis and differentiation [1]. Activating mutations in both genes have been found in colorectal cancer with mutation frequencies of 4-13% for *BRAF *[2-9] and of 20-50% for *K-ras *[10-17] having been reported. *BRAF *and *K-ras *mutations are frequently found to be mutually exclusive in colorectal cancer [5,18] and both genes harbour the majority of mutations in distinct hotspots: *BRAF *at codons 463-468 [19] and 600 [18,19] and *K-ras *at codons 12 and 13 [20] and also, but more infrequently, at codon 61 [21].

Colorectal cancer is the third most common cancer in the world with incidence rates varying up to 25-fold between countries [22]; it has been postulated that approximately 80% of the observed differences in incidence rates between countries can be attributed to dietary factors [23]. Although analysis of dietary components has been performed in relation to incidence of this cancer in general, the exact relationship of dietary factors to specific gene mutations and signalling pathway alterations remains to be fully elucidated. To date, analysis of dietary factors in relation to oncogenically activated ERK pathway members in colorectal cancer has only been performed in a limited number of studies and almost exclusively with regard to *K-ras *mutation. One study reported that low calcium intake and high monounsaturated fat intake were associated with *K-ras *mutated colorectal tumours [24] but this was not confirmed subsequently [14]. Another report linked polyunsaturated fatty acid consumption to *K-ras *mutated colorectal tumours [25]. At present, very few data exist describing *BRAF *mutations in relation to dietary factors, with only one study analysing folate, fibre and alcohol consumption and showing no association between *BRAF *mutation and these dietary constituents [26].

The present study aimed to investigate the relationship between *BRAF *and *K-ras *mutations in 186 adenocarcinomas from the EPIC Norfolk cohort and clinicopathological features, lifestyle and dietary factors. This study is the most comprehensive to date examining dietary factors and *BRAF *mutations in colorectal cancer. Dietary factors which have not been previously tested for association with *K-ras *mutations in colorectal cancer were also explored.

## Methods

### Study population

The EPIC Norfolk cohort included 25 639 healthy men and women aged between 45 and 79 years residing in Norfolk, United Kingdom. Study participants were recruited between 1993 to 1997, from whom information on lifestyle and diet was collected prospectively and who were followed up for incident cancers and other health endpoints. Exact cohort details, blood DNA extraction methods and data collection methods are described in detail elsewhere [27-29]. Ethical approval was obtained from the Norwich Local Research Ethics Committee.

### Case ascertainment and tissue samples

Incident cases of colorectal cancer (International Statistical Classification of Diseases and Related Health Problems (ICD) 9th revision, 153.0-153.9, 154.0, and 154.1) were ascertained by matching study participants to the East Anglian Cancer Registry and Information Centre (ECRiC) in conjunction with data from the United Kingdom Office for National Statistics. Cases of colorectal cancer which developed after the first year following recruitment were used for analysis. As of June 2004, 291 participants in the Norfolk cohort were reported as having been diagnosed with colorectal cancer. For individuals from whom archival tissue was available, formalin-fixed, paraffin-embedded (FFPE) tissue blocks and histopathological reports were obtained from the Norfolk and Norwich University Hospital. Clinicopathological data describing tumour location, Dukes' stage and differentiation were obtained from pathology reports and the ECRiC. Available for this study were 186 adenocarcinoma and 16 adenoma samples from 189 individuals (adenocarcinoma and separate adenoma tissue was obtained from the same individual in 13 cases, adenoma tissue in isolation was resected from 3 individuals and adenocarcinoma tissue in isolation was resected from 173 individuals). The 16 adenomas were either tubular or tubulo-villous adenomas with low grade dysplasia, none displayed significant serrated architecture, and all adenomas were considered together as a single group of pre-malignant neoplasms.

### DNA extraction

FFPE sample blocks were cut into 4 μm sections. One section was stained with haematoxylin and eosin and was used for histological identification of adenoma, adenocarcinoma and normal cell types within the sample. This section was used as a template for isolation of the different cell types from a further five 4 μm sections. Following histological examination, 186 adenocarcinomas and 16 adenomas were identified. Different tissue types were macrodissected and scraped into 240 μl of Buffer PKD with 10 μl of Proteinase K (both obtained from RNEasy FFPE kits, QIAGEN, Valencia, USA). Samples were agitated at 150 rpm at 55.0°C for 4-6 days. Tissue digestion was checked after 3 days and samples which still had visible amounts of tissue had a further 10 μl of Proteinase K added for the remainder of the incubation. Samples were then incubated at 80.0°C for 15 minutes in order to partially reverse formaldehyde modification of the nucleic acids and to denature any residual protein. DNA concentration was then checked using Nanodrop ND-1000 Spectrophotometer (Labtech International Ltd, Ringmer, UK).

### Mutation detection

PCR amplification of the known mutation hotspots of *BRAF *and *K-ras *was performed. *BRAF *exon 11 was amplified using primers 11F (5'-CCT GTA TCC CTC TCA GGC ATA AGG-3') and 11R (5'-GAA CAG TGA ATA TTT CCT TTG ATG-3'). *BRAF *exon 15 was amplified using primers 15F (5'-CTT CAT AAT GCT TGC TCT GAT AGG-3') and 15R (5'-GCA TCT CAG GGC CAA AAA T-3'). PCR products were generated using 5 ng-2 μg of template DNA. KOD Hot Start DNA Polymerase kits (Novagen, Madison, USA) were used to make the following reaction mixture: 2.5 μl × 10 PCR Buffer for KOD Hot Start DNA Polymerase, 1 μl primers, forward and reverse (10 μM each), 1 μl MgSO_4 _(25 mM), 2 μl dNTPs (2 mM each), 0.25 μl KOD DNA Polymerase and made up to a total reaction volume of 25 μl with water. The reactions involved a denaturation step at 94.0°C for 5 minutes followed by 45 cycles of 94.0°C for 15 seconds, 30 seconds at annealing temperatures of 58.1°C and 58.4°C for *BRAF *exons 11 and 15 respectively and 72.0°C for 30 seconds. Final extension was 72°C for 5 minutes. To detect successful amplifications, 5 μl of each reaction mixture was separated on a 3% agarose gel containing 1 μg/ml ethidium bromide, and visualised under UV light.

*K-ras *exon 1 was amplified using previously described primers [30]. *K-ras *exon 2 was amplified using primers 2F (5'-GCA CTG TAA TAA TCC AGA CTG TGT TTC-3') and 2R (5'-GAC AGC TTA TTA TAT TCA ATT TAA AC-3'). The PCR reaction mixture and reaction conditions were as described for *BRAF *except that annealing temperatures of 60.0°C and 55.0°C were used for amplification of *K-ras *exons 1 and 2 respectively. For dideoxysequencing, the remaining PCR amplification product mixture (20 μl), following visualisation on agarose gels, was purified using Multiscreen filter plates (Millipore, Billerica, USA) according to the manufacturer's instructions and subjected to direct sequencing by ABI3730xl Platform sequencer (Applied Biosystems, Warrington, UK). Forward and reverse strands were both sequenced. Every sample was PCR amplified and sequenced a minimum of twice on each strand.

*K-ras *exon 1 was also analysed at codons 12 and 13 with pyrosequencing using a previously described assay which has been shown to be of greater sensitivity than dideoxysequencing when detecting base changes at these positions [31]. Both methods were used to maximise the sensitivity of mutation detection at the highly mutated codons 12 and 13 in exon 1. Following PCR generation of an 82 bp amplicon (reaction mixture as described for *BRAF*, primers and primer annealing temperatures are described elsewhere [31]) reaction mixture was subjected directly to pyrosequencing. Pyrosequencing was performed on two independent PCR products at each of the bases analysed, such that 6 independent reactions were undertaken for each sample (twice at bases 1 and 2 of codon 12 and twice at base 2 of codon 13).

### Microsatellite stability status determination

Determination of microsatellite stability status is described elsewhere (Gay L *et al*., submitted). In brief, six microsatellites were used for microsatellite stability status determination: BAT25, BAT26, BAT40, D2S123, D5S346 and D17S250. PCR primers for amplification of cancer DNA were labelled with 5'6-FAM and primers for amplification of corresponding non-cancerous DNA from the same individual (obtained from blood samples) were labelled with 5'HEX. PCR amplicons covering each marker were analysed for changes in size using an ABI3730xl Platform sequencer with a Genescan 500 ROX size standard (Applied Biosystems, Warrington, UK), and ABI Peak Scanner software (version 1.0). If two or more of the six markers in cancer DNA demonstrated a deviation in size from the same markers analysed in corresponding non-cancerous DNA, the cancer sample was classified as showing microsatellite instability (MSI). If one or none of the markers demonstrated size deviation, the sample was classified as microsatellite stable (MSS).

### Lifestyle and other exposure assessment

Height and weight information were obtained using a baseline health examination upon recruitment between 1993 and 1997 and body mass index (BMI) calculated. Health and lifestyle questionnaires administered at the same time recorded information pertaining to hormone replacement therapy (HRT), smoking habits including smoking status (current, former or never) and pack years of cigarette use (defined as 20 cigarettes a day for a year) and habitual physical activity, assessed using questions referring to present activity at the time of questionnaire administration. Those with low physical activity were defined as those with a sedentary job with no recreational activity, a sedentary job with less than 0.5 hours of recreational activity per day, or a standing job with no recreational activity. High physical activity included those with activity levels above these definitions. Details of the measurements and questionnaires used for attainment and assessment of these data are described elsewhere [28,32,33].

Dietary assessment was performed using seven day food diaries (7dd) which were completed at recruitment. This method of dietary assessment has been previously validated and is described in detail elsewhere [27]. Diaries were completed at recruitment. Food descriptions and portion size estimates were converted into weights of foods and food group data derived. Intakes of meat, fish, fruit and vegetables were assessed as the weights of foods contributing to these food groups. Meat classifications included red, processed and white meats and white and fatty fish types. Red meat was defined as beef, lamb, mutton, pork, veal, rabbit and venison, including composite dishes and excluding offal. White meat was defined as chicken, turkey, duck, guinea fowl, goose, pheasant, grouse and other birds, and as all meat/joints simply cooked or in composite dishes. Processed meat was defined as meat that had undergone smoking, curing, salting or the addition of chemical preservatives. White fish was defined as fish where fat is concentrated in the liver rather than the flesh, such as cod, haddock etc. and fatty fish was defined as fish where fat is distributed throughout the flesh, such as herring and mackerel. Shellfish were not included in either fish category. Fruit consumption was defined as intake of fruits in all forms, including fruit included in composite dishes, but excluding fruit from juices, cereals and jams. Vegetable consumption was defined as intake of vegetables in all forms excluding potatoes, legumes, herbs, pickles and chutneys, and tomato sauces in canned products. Alcohol, fat, vitamin, calcium and macronutrient intakes were calculated using the Data into Nutrients for Epidemiological Research (DINER) program, which is described in detail elsewhere [34].

### Statistical analysis

Analysis of lifestyle and patient characteristics was performed using chi-squared (χ^2^) tests for categorical data and analysis of variance (ANOVA) tests for all continuous numerical data. For this analysis, all 186 adenocarcinomas were classified as *BRAF *or *K-ras *mutated or wildtype. For additional testing, *K-ras *mutated adenocarcinomas were classified by mutation type as either those cancers exhibiting G to A transitions or cancers exhibiting any other mutation type. Clinicopathological cancer features were examined in relation to these mutation categories. Dukes' stage was classified as early Dukes' stage (A and B) and late Dukes' stage (C and D). Tumour location was classified as proximal colonic and distal colonic/rectal. Cancers of other origins (i.e. appendix or secondary metastases) were omitted from location testing. Differentiation was determined by a histopathologist and classified as moderately/well differentiated or poorly differentiated and microsatellite instability status as MSS or MSI. Lifestyle factors, including smoking status (current/former/never), physical activity (high/low), alcohol consumption (g/day, continuous), low-density (mmol/l, continuous) and high-density lipoprotein blood concentrations (mmol/l, continuous), triglyceride blood concentrations (mmol/l, continuous) and plasma vitamin C concentrations (μmol/l, continuous) were also tested for association with the defined mutation categories. Continuous dietary variables were tested for association with mutation including meat, fruit and vegetable, fat, vitamin, and fibre and macronutrient, including calcium, variables, in their relevant unit of consumption. A probability value of less than or equal to 0.05 was considered to be statistically significant. No adjustment was made for multiple testing. All testing was performed using SPSS version 16.0 (SPSS Inc, Chicago, USA).

## Results

### BRAF and K-ras mutation frequencies in colorectal adenocarcinomas and adenomas

**Figure 1:**
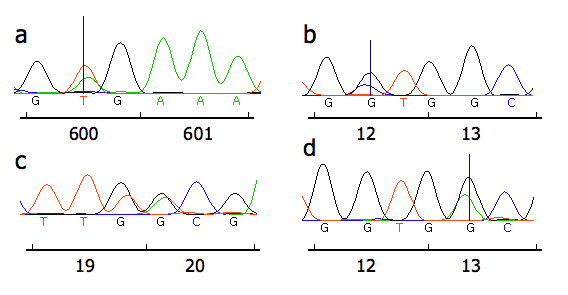
**Colorectal tumour oncogene sequence traces generated using dideoxysequencing**. a: codon 600 valine to glutamic acid change in *BRAF*. b: codon 12 glycine to alanine change in *K-ras*. c: the previously unreported double mutation observed in codons 19 and 20 of *K-ras *resulting in leucine to phenylalanine and threonine to alanine changes at codons 19 and 20 respectively. Additional analyses demonstrated these two base changes to be on the same allele (data not shown). d: codon 13 glycine to aspartic acid change in *K-ras*.

The type and distribution of the mutations observed in *BRAF *and *K-ras *in colorectal neoplasms are described in Table 1 with examples in Figure 1. Of the 186 colorectal adenocarcinoma samples analysed, 29 (15.6%) harboured a mutation in *BRAF*. All *BRAF *mutations were the V600E type due to T to A transversion in exon 15, although a previously reported synonymous SNP (rs56101602) was detected in one adenocarcinoma sample in exon 11. None of the 16 adenomas analysed harboured *BRAF *mutations in either exon 11 or 15.

**Table 1:** The type and distribution of mutations in *BRAF *and *K-ras *in the 186 adenocarcinoma and 16 adenoma tissues available from EPIC Norfolk.

	*BRAF *mutation		
	Mutations in adenocarcinomas	Mutations in adenomas	Total
**Codon 600: wildtype GTG**			
GAG (Val to Glu)	29	0	29

**Total**	29	0	29

	***K-ras *mutation**		

	Mutations in adenocarcinomas	Mutations in adenoma	Total
**Codon 12: wildtype GGT**			
GCT (Gly to Ala)	7	1	8
GTT (Gly to Val)	6	1	7
GAT (Gly to Asp)	14	2	16
TGT (Gly to Cys)	4	1	5
AGT (Gly to Ser)	1	0	1
Undetermined*	1	0	1

**Codon 13: wildtype GGC**			
GAC (Gly to Asp)	6	1	7
TGC (Gly to Cys)	1	0	1

**Codon 19: wildtype TTG**			
GTT (Leu to Phe)**	1	0	1

**Codon 20: wildtype ACG**			
GCG (Thr to Ala)**	1	0	1

**Codon 89: wildtype TCA**			
TTA (Ser to Stop)	0	1	1

**Total**	42	7	49

*: One adenocarcinoma sample was classified as mutated by both sequencing methods but the base change identified was not consistent. Dideoxysequencing identified the base change as G to A, pyrosequencing as G to C. Repeated sequencing using both methods did not resolve this and as such the base change was unclassified. **: The two mutations observed in codons 19 and 20 were in the same adenocarcinoma.

Dideoxysequencing analysis of exons 1 and 2 of *K-ras *identified 30 mutations in the 16 adenoma and 186 adenocarcinoma samples. In addition to those mutations identified with dideoxysequencing, pyrosequencing of *K-ras *codons 12 and 13 demonstrated the presence a further 14 mutations in the colorectal tumour samples analysed, confirming the increased sensitivity of this method. In total, *K-ras *mutations in 41/186 different adenocarcinomas (22.0%) were identified. A previously unreported double mutation observed using dideoxysequencing, at codons 19 and 20 (Figure 1) was not in the region analysed with pyrosequencing. Only 1/30 (3.3%) mutations in codons 12 and 13 identified with dideoxysequencing was not identified with pyrosequencing when subsequently analysed. Thirty-three (78.6%) of the base changes observed in *K-ras *in adenocarcinomas were in codon 12. Seven (16.7%) were in codon 13. Two other base changes (4.8% of total) were observed in codons 19 and 20 of the same cancer.

Of the 16 colorectal adenomas analysed, 7 harboured non-wildtype *K-ras *DNA sequences. Six mutations were identified in exon 1, one, at codon 89, in exon 2. The C to A transversion at the second position of codon 89 produced a stop codon. The remaining 6 mutations in exon 1 were observed to be oncogenically activating base changes in codons 12 and 13. Known oncogenically activating mutations were therefore observed in 37.5% of the adenomas tested. Of the 7 adenoma samples which exhibited non-wildtype *K-ras *sequence, 2 adenomas were identified without accompanying adenocarcinoma. Of the 5 adenomas which were resected from individuals from whom independently presenting adenocarcinoma was also resected, two exhibited an identical mutation in both the adenoma and adenocarcinoma. Three exhibited mutations in the adenoma tissue alone with no evidence of a corresponding mutation in the accompanying adenocarcinoma samples.

Testing of the co-incidence of mutation in *BRAF *and *K-ras *demonstrated that the prevalence of mutation in these two genes were inversely associated (p = 0.009, χ^2^). Fifty-nine adenocarcinomas presented with either *BRAF *or *K-ras *mutation, only one of which harboured both.

### Lifestyle and clinicopathological factors in relation to BRAF and K-ras mutations

Analysis of the distribution of cancers with *BRAF *or *K-ras *mutations according to clinicopathological and lifestyle variables is presented in Table 2. Proximal colonic location, poor differentiation and MSI were all associated with *BRAF *mutation (all p < 0.001). The prevalence of cancers with *BRAF *mutation was higher in females and in individuals with a later age at diagnosis, although these differences failed to reach statistical significance (both p = 0.07).

**Table 2:** Clinicopathological and lifestyle characteristics of colorectal cancer cases by *BRAF *or *K-ras *mutation status and *K-ras *mutated cancers by specific *K-ras *mutation types.

	*BRAF *mutation			*K-ras *mutation			*K-ras *mutation type		
Characteristic	Wildtype *n *= 157^†^	Mutant *n *= 29^†^	*P*^‡^	Wildtype *n *= 145^†^	Mutant *n *= 41^†^	*P*^‡^	G to A *n *= 22^†^	Other *n *= 17^†^	*P*^‡^
**Sex**									
Male	52.9 (83)	34.5 (10)		46.9 (68)	61.0 (25)		63.6 (14)	58.8 (10)	
Female	47.1 (74)	65.5 (19)	0.07	53.1 (77)	39.0 (16)	0.11	36.4 (8)	41.2(7)	0.76
**Age at diagnosis **(years)	70.1 (7.9)	72.9 (5.5)	0.07*	71.1 (7.1)	68.5 (8.9)	0.06*	67.2 (9.4)	69.4 (8.7)	0.46*
**Tumour location**									
Proximal colonic	28.3 (41)	75.0 (21)		33.1 (44)	45.0 (18)		42.9 (9)	52.9 (9)	
Distal colonic/Rectal	71.7 (104)	25.0 (7)	**<0.001**	66.9 (89)	55.0 (22)	0.17	57.1 (12)	47.1 (8)	0.54
**Differentiation**									
Well/Moderate	90.6 (125)	59.3 (16)	*(FET)*	85.8 (109)	84.2 (32)		80.0 (16)	87.5 (14)	*(FET)*
Poor	9.4 (13)	40.7 (11)	**<0.001**	14.2 (18)	15.8 (6)	0.80	20.0 (4)	12.5 (2)	0.67
**Dukes' Stage**									
A/B	54.5 (73)	57.7 (15)		61.9 (78)	29.4 (10)		27.8 (5)	33.3 (5)	*(FET)*
C/D	45.5 (61)	42.3 (11)	0.76	38.1 (48)	70.6 (24)	**0.001**	72.2 (13)	66.7 (101)	1.00
**MSI status**									
MSS	90.8 (129)	42.9 (12)	*(FET)*	78.4 (105)	100.0 (36)		^a^	^a^	
MSI	9.2 (13)	57.1 (16)	**<0.001**	21.6 (29)	0.0 (0)	**0.002**	^a^	^a^	^a^
**BMI **(kg/m^2^)	27.3 (4.3)	26.6 (4.3)	0.41*	27.0 (4.3)	27.9 (4.3)	0.26*	28.1 (4.3)	27.7 (4.7)	0.74*
**Alcohol intake **(g/d)	9.9 (15.3)	5.1 (10.4)	0.11*	8.6 (14.1)	11.1 (16.6)	0.36*	11.1 (18.0)	8.6 (11.1)	0.62*
**Smoking status**									
Current	9.9 (15)	13.8 (4)		11.4 (16)	7.3 (3)		13.6 (3)	0.0 (0)	
Former	46.1 (70)	48.3 (14)		45.7 (64)	48.8 (20)		45.5 (10)	52.9 (9)	
Never	44.1 (67)	37.9 (11)	0.74	42.9 (60)	43.9 (18)	0.75	40.9 (9)	47.1 (8)	0.29
**Physical activity**									
Low	66.7 (104)	69.0 (20)		66.0 (95)	70.7 (29)		63.6 (14)	76.5 (13)	
High	33.3 (52)	31.0 (9)	0.81	34.0 (49)	29.3 (12)	0.57	36.4 (8)	23.5 (4)	0.49
**HRT satatus**									
Current	9.6 (7)	5.3 (1)		9.2 (7)	6.2 (1)		^b^	^b^	
Former	16.4 (12)	21.1 (4)		17.1 (13)	18.8 (3)		^b^	^b^	
Never	74.0 (54)	73.7 (14)	0.78	73.7 (56)	75.0 (12)	0.93	^b^	^b^	^b^
**LDL **(mmol/l)	4.14 (1.1)	4.06 (1.1)	0.75*	4.19 (1.2)	3.91 (0.8)	0.19*	3.85 (0.7)	3.93 (1.0)	0.79*
**HDL **(mmol/l)	1.30 (0.4)	1.33 (0.3)	0.63*	1.33 (0.4)	1.19 (0.4)	0.04*	1.16 (0.4)	1.16 (0.4)	0.96*
**Triglyceride **(mmol/l)	2.11 (1.2)	1.94 (1.1)	0.48*	2.02 (1.1)	2.34 (1.3)	0.12*	2.23 (1.2)	2.59 (1.5)	0.43*
**Plasma vitamin C **(μmol/l)	52.0 (24.3)	48.0 (20.9)	0.44*	52.5 (24.8)	47.5 (20.0)	0.28*	45.4 (19.7)	50.0 (20.9)	0.52*

^‡^P values determined by χ^2 ^test or Fisher's exact test when one or more expected values were less than 5 (denoted by *FET*). *: ANOVA tests used to calculate p-values. Results presented as [*n *(%)] or [mean (± SD)]. HRT: hormone replacement therapy. MSI: Microsatellite instability. MSS: Microsatellite stable. ^† ^Not all individuals had data for each variable, in these cases these individuals were omitted from the test. For HRT testing only females were analysed, for which 92 had data available. ^**a **^Microsatellite instability was not tested relative to different *K-ras *mutation types as none of the cancers harbouring *K-ras *mutation exhibited microsatellite instability. ^**b **^HRT status was not tested relative to different *K-ras *mutation types due to the low numbers of cases available for testing.

A higher proportion of cancers harbouring *K-ras *mutation was found to have later Dukes' stage (C or D rather than A or B)(p = 0.001) and to be microsatellite stable (MSS)(p = 0.002). Of the 36 cancers with available microsatellite stability data and *K-ras *mutation, all were MSS. Cases of cancers harbouring mutated *K-ras *also demonstrated earlier age at diagnosis, although this association failed to reach statistical significance (p = 0.06). Cases with *K-ras *mutation had significantly lower mean blood HDL cholesterol concentrations than those with wildtype *K-ras *(1.19 mmol/l versus 1.33 mmol/l; p = 0.04).

### Dietary factors and BRAF and K-ras mutations

None of the dietary factors tested displayed a statistically significant association with *BRAF *mutations in colorectal cancers. Individuals harbouring *K-ras *mutated cancers had a statistically significantly increased mean white meat consumption: 29.5 g/d *versus *17.4 g/d, p < 0.001 (Table 3).

**Table 3:** Dietary intakes stratified by *BRAF *and *K-ras *mutation status.

	*BRAF *mutation			*K-ras *mutation		
Dietary factor	Wildtype *n *= 156	Mutant *n *= 29	*P*^‡^	Wildtype *n *= 144	Mutant *n *= 41	*P*^‡^
**Meat**						
Red Meat (g/d)	37 (28.9)	40 (24.5)	0.60	38 (28.4)	33 (27.2)	0.31
Processed Meat (g/d)	24 (19.7)	25 (14.3)	0.81	25 (18.7)	23 (19.8)	0.62
Red + Processed Meat (g/d)	61 (37.1)	65 (28.5)	0.59	63 (35.9)	56 (35.4)	0.29
White Meat (g/d)	21 (20.2)	17 (15.5)	0.29	17 (18.0)	30 (22.1)	**<0.001**
White Fish (g/d)	17 (15.7)	17 (26.5)	0.98	18 (19.3)	14 (10.7)	0.28
Fatty Fish (g/d)	12 (20.0)	10 (12.1)	0.64	11 (17.5)	14 (23.4)	0.33
**Fruit and vegetables**						
Fruit (g/d)	170 (133.1)	193 (170.9)	0.42	168 (143.0)	191 (125.8)	0.36
Vegetables (g/d)	136 (68.3)	150 (70.9)	0.29	136 (66.6)	145 (76.1)	0.43
**Fat**						
Total Fat (g/d)	71 (22.9)	71 (23.7)	1.00	71 (22.4)	75 (25.0)	0.32
PUFA (g/d)	13 (5.3)	13 (5.7)	0.96	13 (5.1)	14 (6.2)	0.20
MUFA (g/d)	25 (8.1)	24 (7.5)	0.77	24 (7.9)	26 (8.5)	0.29
SFA (g/d)	27 (10.2)	28 (11.7)	0.84	27 (10.3)	28 (10.7)	0.59
**Vitamins**						
B2[riboflavin] (mg/d)	2 (0.6)	2 (0.6)	0.95	2 (0.6)	2 (0.6)	0.67
B3[niacin] (mg/d)	18 (5.5)	18 (6.7)	0.69	18 (5.9)	19 (4.7)	0.40
B6[pyroxidine] (μg/d)	2 (0.6)	2 (0.6)	0.96	2 (0.6)	2 (0.6)	0.35
B9[folate] (μg/d)	259 (71.9)	257 (73.8)	0.89	258 (73.4)	260 (67.7)	0.89
B12 (μg/d)	6 (5.5)	5 (4.0)	0.40	6 (5.2)	6 (5.4)	0.96
C (mg/d)	85 (48.7)	87 (38.8)	0.82	85 (45.5)	87 (53.4)	0.78
D (μg/d)	3 (2.2)	4 (2.3)	0.69	3 (2.1)	4 (2.6)	0.53
**Fibre and Macronutrients**						
Total Energy (MJ/d)	8 (2.1)	8 (1.8)	0.85	8 (2.1)	8 (2.0)	0.51
Carbohydrate (g/d)	235 (68.2)	250 (58.5)	0.25	238 (68.6)	236 (60.9)	0.87
Protein (g/d)	70 (15.1)	68 (14.8)	0.48	70 (15.4)	72 (13.6)	0.29
NSP (g/d)	14 (5.0)	16 (7.4)	0.15	14 (5.8)	15 (4.1)	0.53
Calcium (mg/d)	779 (235.9)	821 (220.5)	0.37	787 (239.8)	783 (212.7)	0.92

^‡^P values determined by ANOVA. Results presented as [mean (± SD)]. PUFA: polyunsaturated fatty acid. MUFA: monounsaturated fatty acid. SFA: saturated fatty acid. NSP: non-starch polysaccharide. For one case for which mutational status had been determined no dietary data was available. Therefore, for all testing with dietary factors the number of combined cases in the wildtype and mutated groups available for analysis was 185.

### Lifestyle, dietary and clinicopathological factors of colorectal cancers in relation to K-ras mutation type

Of the 42 *K-ras *mutations observed in colorectal cancers, 22 were G to A transitions. In order to assess the relevance of this observation, individuals with *K-ras *mutated cancers exhibiting this specific G to A base change were compared with individuals harbouring *K-ras *mutated cancers with all other mutation types such that *K-ras *mutations were classified as 'G to A' or 'other'. One sample, harbouring a double mutation at codons 19 and 20 was omitted from the testing due to the base changes being of both classifications. A second sample was classified as mutated by both dideoxysequencing and pyrosequencing methods. However, the two methods described a G to A and a G to C base change respectively, confirmed following repeated testing. This case was also omitted from this analysis. Analysis of the distribution of cancers with different *K-ras *mutation types according to clinicopathological and lifestyle variables is summarised in Table 2: none of the clinicopathological features or lifestyle exposures tested were associated with either classification of *K-ras *mutation.

All dietary variables were tested for association with either *K-ras *mutation classification. Cancers harbouring G to A transitions in *K-ras *were found in individuals with a significantly lower consumption of fruit compared with those individuals harbouring other *K-ras *mutation types (p = 0.02): 155 g/d *versus *247 g/d. A reduced consumption of vegetables was also observed in those individuals with *K-ras *G to A base changes in their cancers, although this did not reach statistical significance (p = 0.07).

## Discussion

The data presented herein suggest that *BRAF *and *K-ras *mutations arise in an almost mutually exclusive manner in distinct subsets of colorectal cancer, that differ in terms of clinicopathological features, dietary factors and lifestyle exposures. *BRAF *mutation in colorectal cancers was observed at a frequency of 15.6% and *K-ras *mutation at 22.0%. These frequencies are at the high and low ends of the ranges previously reported for mutations in these genes in colorectal cancer: 4-13% for *BRAF *[2-9] and 20-50% for *K-ras *[10,12-17,35]. The mutually exclusive nature of these two mutation types, as shown in this and other studies [3,5,7,18,36] may explain this: increased prevalence of *BRAF *mutation in this sample set may be consistent with a reduction in *K-ras *mutation frequency.

*BRAF *mutation was strongly associated with cancer of proximal colonic location, poor differentiation and microsatellite instability (all p < 0.001). *BRAF *mutation and proximal colonic location have been linked in previous reports [5]. Microsatellite instability has been linked to proximal location [37] and has been consistently linked to *BRAF *mutation in colorectal cancer [3,4,6]: one review described *BRAF *mutation as a 'hallmark' of MSI tumours [38]. Poor differentiation has also been linked to *BRAF *mutation in previous studies [39,40] as well as microsatellite instability [41]. Taken together, these data confirm that the clinicopathological signature of *BRAF *mutated colorectal cancer includes proximal location, microsatellite instability and poor differentiation. This is consistent with this distinct subset of tumours arising by a mechanism of microsatellite instability, which is strongly associated with *BRAF *mutation. In contrast to this, analysis of *K-ras *mutations demonstrated an association with microsatellite stability (p = 0.002); an observation reported previously [4,6,42]. In this report *K-ras *mutation was more prevalent in cancers of later Dukes' stage (C and D). This observation has been made in some previous reports [13,15] but not in others [2,11,12,17,20,35,43]. Additionally, the largest study to date on *K-ras *mutations in colorectal cancer, analysing 4268 cases, reported an association between *K-ras *mutation in colorectal cancer and poor prognosis [44], suggesting an association between *K-ras *mutation and more advanced colorectal cancer. The testing presented herein demonstrates the independent clinicopathological nature of colorectal cancers with either *BRAF *or *K-ras *mutations.

Analysis of dietary factors stratified by colorectal cancer gene mutation type showed that none of the dietary factors tested were positively or negatively associated with *BRAF *mutation. This study is the first to undertake a comprehensive analysis of *BRAF *mutations in colorectal cancer and their relationship to dietary factors. One previous report analysed folate, alcohol and fibre consumption and found no association with *BRAF *mutation [26], observations which were confirmed in this study. The detailed analysis presented here has demonstrated further the independence of *BRAF *mutation relative to dietary intakes using a comprehensive analysis of twenty four individual dietary constituents.

Analysis of *K-ras *mutations in colorectal cancers in relation to dietary factors demonstrated that mutation in *K-ras *was associated with increased white meat consumption (p < 0.001). Furthermore, analysis of *K-ras *mutation type identified those individuals harbouring *K-ras *mutated cancers with G to A transitions as consuming less fruit (p = 0.02) and vegetables, although the reduction in vegetable consumption was of marginal statistical significance (p = 0.07). Individuals harbouring *K-ras *mutated cancers also had lower blood HDL cholesterol concentrations than those harbouring cancers with wildtype *K-ras *genes (p = 0.04), an association requiring validation in future studies. The association between increased white meat consumption and *K-ras *mutations in colorectal cancer requires confirmation in larger studies and *in vitro *mechanistic investigations. However, plausible mechanisms have been postulated which may explain reduced fruit and vegetable consumption and increased prevalence of G to A transitions. Fruits and vegetables contain bioactive compounds, such as flavanols, capable of inhibiting nitroso compound formation [45]. Nitroso compounds are capable of inducing guanine base alkylation which, if not repaired, can lead to G to A base transitions [46]. Therefore, low fruit and vegetable consumption is consistent with G to A transitions in tumours, as demonstrated in this study. This study did not confirm observations made in a previous study of *K-ras *mutated colorectal tumours being associated with low calcium and high monounsaturated fat intake [24]. These observations were also tested in another large population based study, which also failed to detect this association [14]. Another finding linking polyunsaturated fatty acid types to *K-ras *mutated tumours [25] was also not confirmed in the data presented here.

In addition to the 186 cancer samples analysed, 16 unselected adenomas were also tested for mutation in *BRAF *and *K-ras*. No *BRAF *mutations were observed in any of the adenomas. A previous analysis of 113 unselected sporadic colorectal adenomas detected *BRAF *mutations at a frequency of only 2.8% [8]. However, in serrated adenomas mutation frequencies for *BRAF *of 30 to 50% of cases have been described [36,47]. Because of the low number of adenomas available for analysis and the lack serrated adenomas in this sample set (none were included), the observation that none of the 16 adenomas tested harboured *BRAF *mutation was expected.

In contrast to *BRAF*, 6 (37.5%) adenomas harboured oncogenically activating mutations in *K-ras*, a prevalence consistent with previous reports [8,48]. In addition to this, a novel *K-ras *mutation was observed in a single adenoma. This mutation was not observed in the corresponding cancer tissue. This point mutation in codon 89 produced a stop codon towards the end of exon 2. This mutation is uncharacteristic of proto-oncogene transformation in that it would not lead to increased ERK signalling. However, the tumour suppressor function of wildtype KRAS protein has been previously described [49] and as such a putative cancer promoting effect of such a truncating mutation in *K-ras *cannot be discounted.

Although the number of adenomas tested was too low to elucidate meaningful relationships between clinicopathological features, lifestyle exposures and dietary intakes, it is interesting to note the lack of *BRAF *mutations observed compared to the relatively high prevalence of oncogenically activating mutations observed in *K-ras *(37.5%). These observations are consistent with the later timing of *BRAF *mutation in colorectal cancer development, which has been postulated previously [50], and is consistent with distinct subsets of colorectal tumours with *BRAF *and *K-ras *mutations.

A strength of the current study is the use of prospective dietary and lifestyle data collected before the onset of disease as well as the use of 7dd records for dietary assessment, a method that has been shown to estimate diet more accurately than food frequency questionnaires when validated with urinary biomarkers [51]. Furthermore, such detailed dietary analysis regarding so many variables has not been previously attempted in relation to mutation of these genes, and as such this report contributes new information to the current knowledge of mutations in *BRAF *and *K-ras *in colorectal cancer and dietary intakes. The limitations inherent in our study of multiple statistical testing mean that the significant associations observed in this study would benefit from further validation. Adjustment for confounding variables in the statistical testing was not performed in this study. It has previously been described how logistic regression analyses performed on low sample sets leads to systematic bias (i.e. away from null), and overestimation of odds ratios [52]. Consequently, in order to prevent overestimation of dietary risk factors, this testing was not performed on the small sample sizes available. Further validation of the exploratory associations presented in this report would benefit from testing in studies with larger sample sets upon which confounding factors could be extensively explored.

## Conclusions

*BRAF *mutations are found in cancers with a clinicopathological signature of proximal colonic location, poor differentiation and microsatellite instability. Furthermore, the presence of *BRAF *mutations in colorectal cancer is not associated with any of the dietary factors tested. Conversely, *K-ras *mutations are not characteristic of colorectal cancers with these clinicopathological features and are found in microsatellite stable colorectal cancers and are associated with a more advanced Dukes' stage. Unlike *BRAF *mutations, *K-ras *mutations, in general and specific types, appear to be associated with specific dietary factors. These data demonstrate the independent distribution of *BRAF *and *K-ras *mutations in different subsets of colorectal cancer.

## Competing interests

The authors declare that they have no competing interests.

## Authors' contributions

AN performed the sequencing analyses, statistical testing and composed the manuscript. PNM contributed to manuscript preparation and directed the display of statistical data. LJG performed the MSI analyses. RYB obtained access to and distributed the human tissue samples. RNL and AM compiled and provided the dietary data. MJA contributed to study design, histopathological analysis of the samples, manuscript preparation and supervision of the project. JCC and SAR contributed to study design and coordination. All authors read and approved the manuscript, except SAR. Sheila Rodwell's initial contributions were of great help and guidance during the concept and initiation stages of this study. With great regret, Sheila's untimely passing in June 2009 meant that she was unable to witness the outcome of the research described here.

## Pre-publication history

The pre-publication history for this paper can be accessed here:

http://www.biomedcentral.com/1471-2407/10/99/prepub
